# Nurse-led mobile health-based nudging intervention program to support exercise behavior among community-dwelling frail older adults: A multiphase intervention development study

**DOI:** 10.1016/j.ijnsa.2026.100690

**Published:** 2026-09-24

**Authors:** Ruotong Peng, Jia Yu, Ronghua Yang, Hui Feng, Yinan Zhao

**Affiliations:** a School of Nursing, Chinese Academy of Medical Sciences & Peking Union Medical College, Beijing, China; b Xiangya School of Nursing, Central South University, Changsha, China; c Department of Neonatology, The First Affiliated Hospital of Hunan College of Traditional Chinese Medicine, Zhuzhou, China

**Keywords:** Nudge, Nudging intervention, Technology, Mobile health, Exercise, Older adults, Intervention development

## Abstract

**Background:**

Multicomponent exercise is widely recommended to prevent and delay frailty. Despite the health benefits of exercise, participation in and adherence to exercise among community-dwelling frail older adults remain suboptimal.

**Aim:**

To develop a nurse-led mobile health-based nudging intervention program to support exercise behavior among community-dwelling frail older adults.

**Methods:**

This intervention development study was informed by the Guidance for Reporting Intervention Development Studies in Health Research checklist. The study comprised four sequential development phases followed by digital translation for delivery: scoping review, semi-structured interviews, theory-informed development, and structured expert consultation. The finalized intervention program was then translated into digital delivery.

**Results:**

A 12-week nurse-led mobile health-based nudging intervention was developed. The intervention program was grounded in the Capability, Opportunity, Motivation–Behavior model and targeted frail older adults' physical and psychological capability, physical and social opportunity, and reflective and automatic motivation for exercise. Fourteen nudge strategies were mapped to intervention components, including priming, information support, simplification, default options, emotional anchoring, feedback, expert authority, environmental cues, social norms, precommitment, framing, reminders, peer comparison, and gamification. These strategies were operationalized into intervention components, including technical guidance, health education, exercise lessons, future self-visualization, psychological adaptation, environmental modification, professional support, peer support, self-monitoring, goal setting, framing reminders, role modeling, and gamification. The final program specifies behavioral targets, nudge strategies, intervention content, delivery stages, and delivery platforms.

**Conclusion:**

The program was developed through a theory-, evidence-, participant-, and expert-informed process and may provide a structured intervention prototype for supporting exercise behavior among community-dwelling frail older adults. As this was a multiphase intervention development study, future pilot studies and randomized controlled trials are needed to evaluate implementation feasibility, user acceptability, intervention effectiveness, and optimal combinations of nudge strategies.

**What is known?**

 

• Multicomponent exercise is recommended for preventing and delaying frailty, but participation and adherence remain difficult for community-dwelling frail older adults.
• Mobile health can extend exercise support into home settings, but digital exclusion, low digital literacy, and limited technical support may reduce usability among frail older adults.
• Nudging can reduce cognitive and motivational barriers to health behavior, yet it has rarely been operationalized in mobile exercise interventions tailored to frail older adults.

**What does this paper add?**

 

• This study developed a nurse-led, 12-week mobile health-based nudging intervention program through evidence synthesis, qualitative interviews, theory-informed development, structured expert consultation, and digital translation.
• The intervention explicitly maps capability, opportunity, and motivation determinants to nudge strategies and concrete intervention components.
• The study provides a transparent development pathway for translating behavioral determinants and nudge strategies into nurse-led, low-burden, digitally supported components that can be further refined and evaluated in community nursing practice.

## 1 Introduction

Frailty, characterized by age-associated physiological decline, poses severe health risks and creates a substantial burden on healthcare systems (Chen et al., 2014; Kwak and Thompson, 2021). Globally, the prevalence of community-dwelling frailty and pre-frailty is 9.9% and 44.2%, respectively (Ofori-Asenso et al., 2019). This burden is particularly acute in China, where the prevalence of frailty and pre-frailty is 12.7% and 47.3%, respectively, leading to a 1.54-fold increase in the demand for long-term care (Clegg et al., 2013). While multicomponent exercise, such as the VIVIFRAIL program, is widely recommended as a first-line intervention to delay frailty (Dent et al., 2019; Izquierdo et al., 2025; Sánchez-Sánchez et al., 2022), its real-world implementation faces significant challenges, including limited accessibility, low participation, and poor compliance. Most older adults engage in monotonous routines, primarily low-intensity activities like walking. As demonstrated by Yu et al. (2025), the participation rate of regular exercise among frail older adults was only 29.1%, with poor adherence. The intention-behavior gap further compounds this low engagement. Although rationally aware of the health benefits of exercise, older adults often prioritize immediate sedentary comfort over long-term rewards (Feil et al., 2023; Peng et al., 2025). Sustained exercise depletes self-regulatory capacity, while pre-existing unhealthy habits may compound this depletion and further undermine exercise adherence. To address these issues, effective behavioral strategies should be developed to bridge the intention-behavior gap and promote sustained exercise.

Nudging interventions may support exercise behavior by altering choice architecture without mandating a particular choice. Compared with behavior change approaches that primarily target reflective motivation and self-regulation, nudging approaches start from actual decision-making patterns and seek to address cognitive biases such as availability bias, loss aversion, overconfidence, and optimism bias (Beyera et al., 2022; Kwasnicka et al., 2016; Phillips et al., 2024). This perspective is broadly consistent with dual-process accounts of decision-making, in which behavior is shaped by both automatic and reflective processes. In the context of exercise promotion, nudging strategies may reduce the cognitive and practical effort required to initiate and maintain activity by simplifying choices, embedding environmental cues, increasing the salience of exercise-related information, providing timely reminders and feedback, activating precommitment, and drawing on social norms (Thaler, 2018; Thaler and Sunstein, 2008; Zhang et al., 2021). However, nudge-based exercise interventions specifically designed for community-dwelling frail older adults remain limited. Combining the Capability, Opportunity, Motivation–Behavior model with nudge strategies may add value by linking behavioral diagnosis with choice-architecture design: the Capability, Opportunity, Motivation–Behavior model helps identify what needs to be supported, whereas nudge strategies help determine how support can be embedded into daily exercise decisions with minimal burden.

Mobile health interventions can extend exercise support into home settings and reduce barriers related to weather, geography, and time flexibility, particularly for older adults with limited access to community-based services (McGarrigle and Todd, 2020; Soto-Bagaria et al., 2023). The World Health Organization defines mobile health as "medical and public health practice supported by mobile devices, such as mobile phones, monitoring devices, digital assistants, and other wireless devices" (Kay et al., 2011). In terms of behavioral nudge, mobile health technology enables automated reminders, personalized feedback, and enhanced incentives, reducing reliance on professionals and lowering the cost of interventions (Galway et al., 2023; Lee et al., 2023; Li et al., 2024). Existing mobile health-based exercise interventions for frail older adults commonly include exercise prescriptions, home-based exercise videos, activity monitoring, reminders, and remote feedback (Kwan et al., 2020; Lee et al., 2025). Although these components can improve access to exercise support, they may less explicitly target the daily behavioral processes involved in initiating and maintaining exercise. In this study, mobile health interactions were designed to incorporate nudge strategies that reduce friction, increase the salience of exercise cues, and make exercise easier to initiate.

Therefore, this study aimed to develop a nurse-led mobile health-based nudging intervention program to support exercise behavior among community-dwelling frail older adults.

## 2 Methods

### 2.1 Study design

This study was defined as a multiphase intervention development study. Reporting of the intervention development process was informed by the Guidance for Reporting Intervention Development Studies in Health Research checklist (Duncan et al., 2020), which emphasizes the transparent description of the rationale, evidence base, theory, stakeholder involvement, development process, intervention refinement, and remaining uncertainties. A completed checklist is provided in Supplementary Table 1.

The study employed four sequential development phases (Fig. 1). Phase 1 consisted of a scoping review to map existing evidence on mobile health-based exercise interventions for older adults, with particular attention to exercise prescriptions, behavioral strategies, theoretical foundations, and outcome evaluation methods. Phase 2 involved semi-structured qualitative interviews with community-dwelling frail older adults to explore their needs, preferences, perceived barriers, and expectations regarding a mobile health-based exercise intervention. Phase 3 involved theory-informed development, in which findings from the scoping review and qualitative interviews were mapped to the Capability, Opportunity, Motivation–Behavior model and nudge strategies to develop the preliminary intervention program. Phase 4 involved structured expert consultation to review and refine the preliminary intervention program. After the final intervention program was determined, digital translation was conducted to operationalize the program into platform-based, complementary delivery materials for future implementation.

**Fig. 1:**
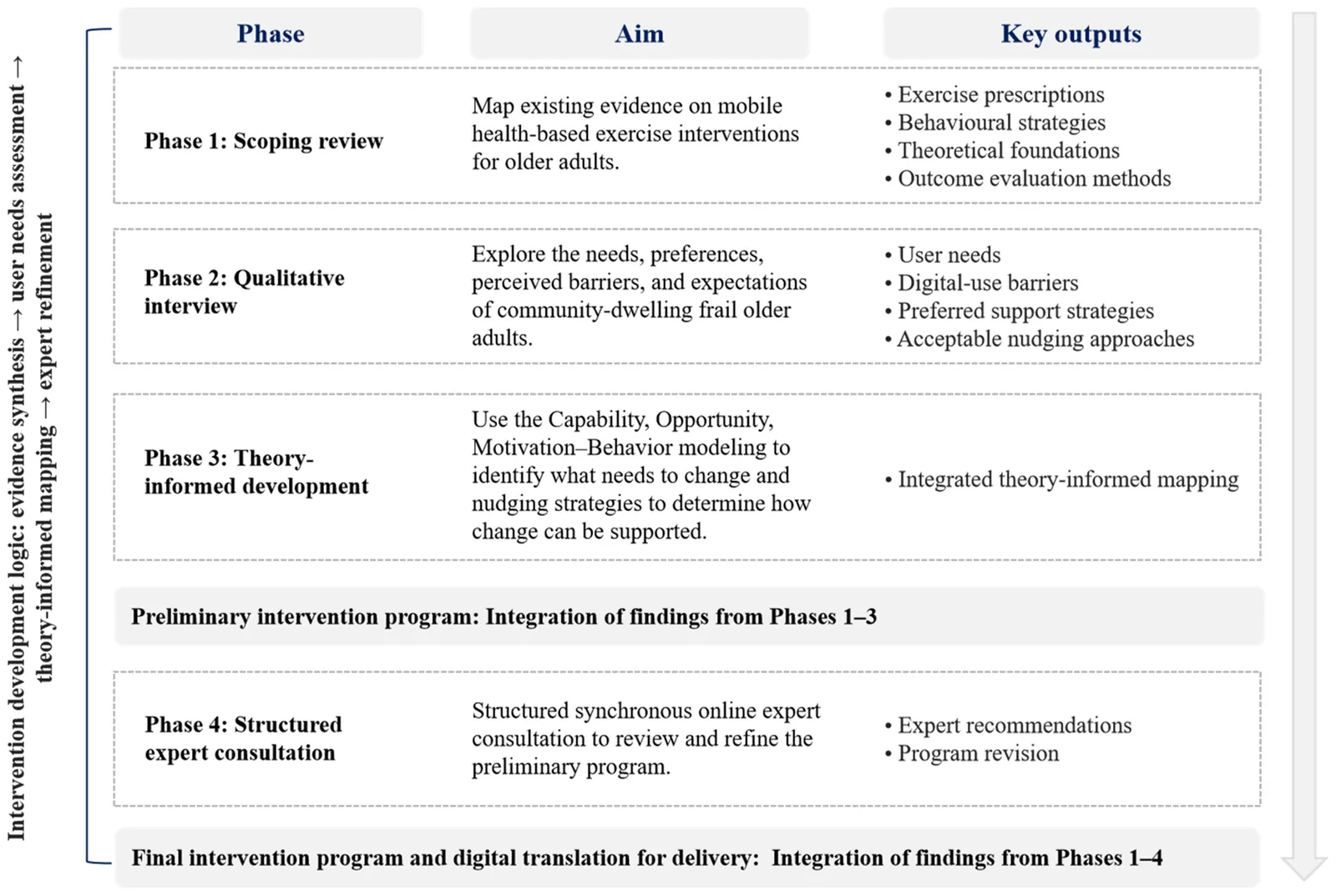
Multiphase development process of the nurse-led mobile health-based nudging intervention program. Note: The program was developed through four sequential steps: scoping review, qualitative interviews, theory-informed development, and structured expert consultation. The finalized program was then translated into digital and complementary delivery materials for future implementation.

### 2.2 Theoretical foundation

This study adopted a dual theoretical approach by integrating the Capability, Opportunity, Motivation–Behavior model with nudge strategies to guide intervention development (Fig. 2). The Capability, Opportunity, Motivation–Behavior model was used to conceptualize exercise participation among frail older adults in terms of capability, opportunity, and motivation, while nudge strategies provided a practical approach for supporting behavior through low-burden changes to the choice environment.

**Fig. 2:**
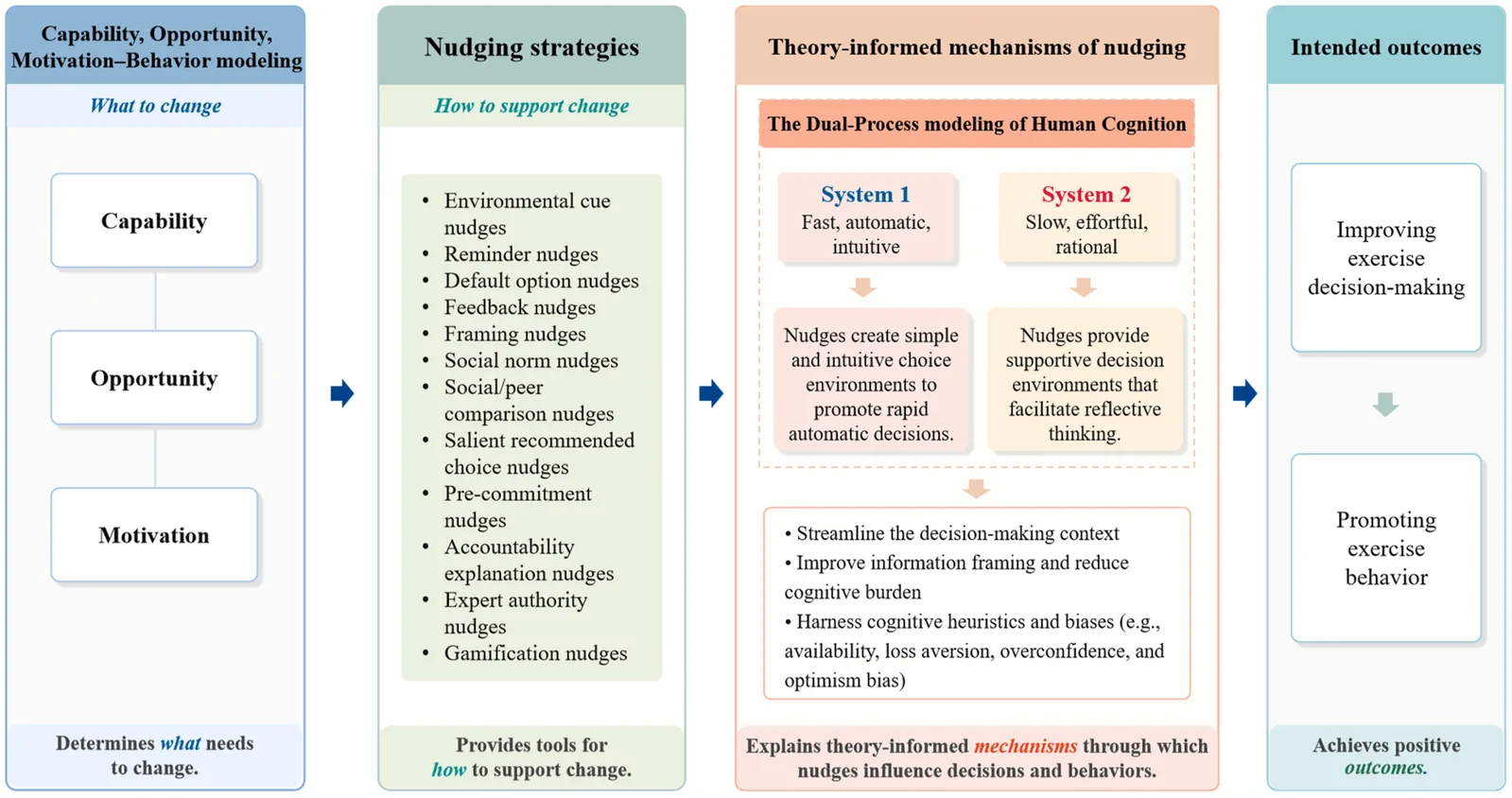
Theoretical framework for integrating the Capability, Opportunity, Motivation–Behavior model and nudge strategies. Note: The Capability, Opportunity, Motivation–Behavior model was used to identify behavioral determinants of exercise participation, whereas nudge strategies were used to guide choice-architecture design and support exercise decision-making among frail older adults.

### 2.3 Scoping review

#### 2.3.1 Research team

The scoping review was conducted in accordance with methodological guidance for scoping reviews and reported using the Preferred Reporting Items for Systematic Reviews and Meta-Analyses extension for Scoping Reviews. The review was conducted by a four-member research team, including one professor in community and geriatric nursing, one rehabilitation therapist, and two master's students. The professor was responsible for overall study supervision and quality control; the rehabilitation therapist contributed professional oversight of exercise-related content; and the two master's students were responsible for literature searching, study screening, data extraction, and data organization.

#### 2.3.2 Identification of research questions

Based on the participant, intervention, context, and outcome framework, the research questions were: (1) What is the current status of mobile health-based nudging intervention programs among frail older adults? (2) What theories have been applied to exercise behavior interventions for older adults? (3) What are the exercise prescriptions, nudge strategies, and methods of evaluating the effectiveness of existing intervention programs? (4) What are the gaps and challenges in current research, and what directions should future research prioritize?

#### 2.3.3 Search strategy

Two graduate students (R.P and J.Y) reviewed the literature. PubMed, Web of Science, Embase (OVID), CINAHL, and the Cochrane Library (five English databases), as well as China National Knowledge Infrastructure, Wanfang Data, and SinoMed, were searched from database inception to March 2024. Search terms combined controlled vocabulary and keywords related to mobile health, exercise or physical activity, older adults, and frailty. The full search string for each database is provided in Supplementary Table 2.

#### 2.3.4 Inclusion and exclusion criteria

Inclusion criteria were as follows: (1) participants were frail or prefrail older adults; no strict age cutoff was applied, and studies including adults aged 55 years or older, 60 years or older, or 65 years or older were eligible because age thresholds for older adult interventions vary across countries and the review focused on intervention components rather than age-specific effect estimation; (2) interventions were delivered through mobile or digital health technologies, including smartphones, wearable devices, tablet computers, or other remote platforms; and (3) randomized controlled trials, quasi-experimental studies, mixed-methods studies, qualitative studies, design studies, feasibility or pilot studies, peer-reviewed protocols, and academic dissertations were eligible. Protocols were included because this review aimed to identify intervention components and design features.

Exclusion criteria were as follows: (1) registered trials, conference abstracts, and reviews; and (2) studies focused solely on mobile health tools for frailty assessment without intervention components.

#### 2.3.5 Study selection and data extraction

EndNote X21 was used to manage the retrieved records. Two authors (R.P. and J.Y.) independently screened titles and abstracts according to the eligibility criteria and then reviewed full texts. Disagreements were resolved through discussion with a third author (Y.Z.). A standardized extraction form was used to collect author, year, country, study design, theoretical framework, delivery platform, intervention characteristics, behavioral strategies, and outcome evaluation methods. Two authors (R.P and J.Y) independently conducted data selection and extraction. Disagreements were resolved through consultation with a third author (Y.Z). A formal methodological quality appraisal was not conducted because the scoping review aimed to map intervention components, theories, and delivery features rather than to synthesize effect sizes or assess intervention effectiveness. However, key design features and outcome evaluation methods were extracted to inform the development of the intervention.

### 2.4 Qualitative interviews

Qualitative interviews were conducted to explore the needs and preferences of community-dwelling frail older adults for a mobile health-based nudging intervention program in Changsha, China, from April to May 2024. Participants were recruited from community settings using purposive sampling, with snowball sampling used where appropriate. Sampling aimed to include older adults with variation in age, sex, frailty status, previous exercise experience, educational background, socioeconomic status, and experience with mobile technologies. The sample size was not predetermined; recruitment continued until data saturation was reached. Saturation was assessed iteratively during concurrent data collection and analysis by the research team. Data saturation was used as the stopping criterion and was defined as the point at which interviews no longer generated new information relevant to user needs, perceived barriers, preferences for mobile health delivery, or acceptable nudge strategies. The qualitative component of this multiphase study has been published separately (Peng et al., 2025).

The interview guide was informed by nudge theory (Thaler, 2018; Thaler and Sunstein, 2008), and covered 14 questions across six areas: prior experience, willingness to use, technical assistance, interface design, functionality requirements, and nudges (Supplementary Table 3). Interviews were audio-recorded with participants’ permission, transcribed verbatim, and checked against the recordings for accuracy. The transcripts were analyzed using a hybrid deductive–inductive thematic analysis. In the deductive stage, initial coding was guided by nudge theory and the Capability, Opportunity, Motivation–Behavior model. In the inductive stage, the researchers remained open to new codes emerging from participants’ accounts that were not fully captured by the initial theoretical framework.

Two researchers independently read the transcripts, generated initial codes, compared coding results, and discussed discrepancies until agreement was reached. Codes with similar meanings were grouped into categories and themes. A senior researcher reviewed the coding framework, category development, and theme interpretation to enhance researcher triangulation and analytical credibility. Key qualitative reporting items were described with reference to the Consolidated Criteria for Reporting Qualitative Research (Tong et al., 2007). Details of the qualitative methods and findings are provided in the separately published qualitative study (Peng et al., 2025).

### 2.5 Theory-informed development of the preliminary intervention program

Findings from the scoping review and qualitative interviews were integrated to develop the preliminary intervention program. The scoping review informed the selection of exercise prescriptions, delivery modes, behavioral support strategies, and outcome evaluation methods, while the qualitative interviews provided information on frail older adults’ perceived barriers, needs, preferences, and acceptable nudging approaches. Development-relevant findings were mapped onto the Capability, Opportunity, Motivation–Behavior domains, and candidate nudge strategies were selected by considering the corresponding behavioral determinants, available evidence, user preferences, acceptability, and feasibility for mobile health-based home delivery. The selected strategies were then translated into concrete intervention components. An integrated mapping table was prepared to link evidence from the scoping review, user needs and preferences from qualitative interviews, Capability, Opportunity, Motivation–Behavior domains, selected nudge strategies, resulting intervention components, and the logic for matching.

### 2.6 Expert consultation for intervention refinement

A structured synchronous online expert consultation meeting was conducted via videoconference to review and refine the preliminary intervention program. Previous complex intervention studies have used expert consultation to appraise evidence, incorporate professional judgment, identify intervention priorities, and refine intervention content (Carter et al., 2025; Leon et al., 2021). The present study used an online expert consultation meeting to refine an already developed intervention prototype through interactive multidisciplinary discussion, rather than to establish a formal consensus statement, clinical guideline, or rating-based indicator system.

#### 2.6.1 Identification of consulting experts

Experts were purposively selected according to five criteria: (1) holding at least a mid-level professional title; (2) having at least five years of relevant clinical, nursing, rehabilitation, research, or software-related experience; (3) having knowledge of nudge strategies or health behavior intervention; (4) being familiar with mobile health-based exercise interventions or community-based service delivery; and (5) being willing to participate actively in the consultation. Experts from clinical medicine, nursing, rehabilitation medicine, and software development were invited to provide multidisciplinary input.

#### 2.6.2 Implementation process of the expert consultation

Before the consultation, experts received written materials, including the study background, objectives, theoretical basis, scoping review findings, qualitative findings, and the preliminary intervention program. During the meeting, the research team first presented the rationale and development process of the program. Experts then discussed each intervention component, focusing on relevance, clarity, safety, acceptability, feasibility, and suitability for frail older adults in community and home settings. Key discussion topics included intervention timing, the distinction between exercise prescriptions and behavioral support strategies, the appropriateness of selected nudge strategies, the role of nurses and other professionals, and the practical delivery of the intervention.

#### 2.6.3 Documentation and program revision

The consultation was documented through meeting notes and field records. After the meeting, expert comments were summarized by topic and used to revise the preliminary intervention program. The revised version was returned to the experts to check whether their comments had been accurately understood and appropriately incorporated. This process supported program refinement before finalization, while expert agreement was described qualitatively rather than quantified.

### 2.7 Digital translation of the final intervention program for delivery

After the final intervention program had been determined, the research team translated the intervention components into digital and complementary delivery materials for future implementation. This step was intended to operationalize the finalized intervention program for delivery, rather than to evaluate the feasibility, usability, acceptability, or effectiveness of the digital platform.

A multidisciplinary development team was established to support the digital translation process, including researchers in community and gerontological nursing, graduate students, a user interface designer, software engineers, and a hardware engineer. The team was responsible for functional requirement analysis, interface design, and system development.

The digital translation process was guided by three principles: user-centered design, consistency, and simplicity. First, user-centered design was used to ensure that the functional and interface design was suitable for community-dwelling frail older adults. Second, consistency was emphasized in concepts, procedures, appearance, layout, and interaction patterns. Third, simplicity and ease of use were prioritized by reducing information load, minimizing operational steps, using concise and familiar terminology, and avoiding complex gestures. These principles were used to support age-friendly digital delivery and reduce the operational burden of using the home-based exercise system.

### 2.8 Ethical considerations

This study received ethical approval from the Institutional Review Board of Xiangya School of Nursing, Central South University (IRB No E202463). Before qualitative data collection, informed consent was obtained from all participants involved. Consent was also obtained from all experts before the expert consultation. The study followed the ethical principles set out in the World Medical Association Declaration of Helsinki (World Medical Association, 2013). Because the planned intervention involved nudging strategies, the program was designed to preserve participant autonomy through transparent explanations of the purpose of reminders, framed messages, feedback, peer interaction, and goal setting. Participation in interactive components would remain voluntary. Emotional anchoring and loss-framing messages were designed as transparent health education prompts rather than coercive messages. Secure storage of personal data and exercise-related records was also required for future implementation.

## 3 Results

### 3.1 Scoping review

#### 3.1.1 Study selection

The initial search yielded 2350 articles. After removing 645 duplicates, 1705 records were screened by title and abstract. Forty-two articles underwent full-text screening, and 14 articles were included (Geraedts et al., 2017; Geraedts et al., 2021; Hong and Kong, 2023; Kerstiens et al., 2023; Meredith et al., 2023; Okpara et al., 2023; Villa-García et al., 2023; Wang et al., 2022; Weiss et al., 2023; Yan, 2021; Yoo et al., 2023; Zhang, 2023; Zhang et al., 2022; Zhu et al., 2021). The study selection process is summarized in the Preferred Reporting Items for Systematic reviews and Meta-Analyses flow diagram (Supplementary Fig. 1).

#### 3.1.2 Study characteristics

A total of 14 studies from China (N = 4), Canada (N = 3), Korea (N = 2), the Netherlands (N = 2), the United States (N = 1), Spain (N = 1), and the United Kingdom (N = 1) were included in the analysis of the included literature. The publication years ranged from 2017 to 2023, with a major focus on 2023. The study designs included feasibility studies (N = 3), randomized controlled trials (N = 2), dissertations (N = 2), quasi-experimental study (N = 1), cross-sectional study (N = 1), design study (N = 1), pilot study (N = 1), co-design study (N = 1), randomized controlled trial protocol (N = 1), and quasi-experimental study protocol (N = 1). Intervention delivery covered mobile health apps (e.g., ViviFrail APP and WeChat Mini Program, a lightweight sub-application within the WeChat ecosystem), wearable devices (e.g., smart insoles, Apple Watch), Zoom remote video tool, and virtual reality platforms. Exercise type was dominated by multicomponent exercise (N = 9), followed by resistance training (N = 3). The basic characteristics of the included literature are detailed in Supplementary Table 4.

#### 3.1.3 Theoretical foundations

The included studies used heterogeneous theoretical foundations. One study adopted the Technology Acceptance Model integrated with the Health Belief Model; one used the Health Belief Model alone; one applied the Behavior Change Wheel; and one implemented the transtheoretical model. However, 10 studies did not specify a theoretical framework and relied primarily on empirical strategies, such as reminders and monitoring, without systematic behavioral explanations. This theoretical heterogeneity indicates a gap in the explicit use of behavior change theories to guide mobile exercise interventions for frail older adults.

#### 3.1.4 Exercise behavior intervention strategies

Existing studies use a variety of behavioral strategies, including technical support, health education, goal setting, reminders, social support, feedback, visual cues, and progress tracking. Although these studies did not explicitly adopt nudge theory, several intervention strategies were consistent with nudge mechanisms. Based on Thaler and Sunstein's classic definition (Thaler and Sunstein, 2008) and the Messenger, Incentives, Norms, Defaults, Salience, Priming, Affect, Commitments, and Ego framework (Dolan et al., 2012), the behavioral strategies identified in the review were considered when selecting candidate nudge strategies for the preliminary intervention program.

### 3.2 Qualitative interviews

Overall, 14 community-dwelling older adults with pre-frailty or frailty were interviewed. Participants were aged 60 to 82 years, with a median age of 64 years. Data saturation was reached during the qualitative phase. The research team judged that no new development-relevant information was emerging in the later interviews, supporting the adequacy of the sample for identifying development-relevant needs, perceived barriers, preferences for mobile health delivery, and acceptable nudge strategies. As the full qualitative analysis has been reported separately (Peng et al., 2025), this section summarizes the findings that directly informed the design of the intervention components and nudge strategies.

The interviews identified several barriers that could limit engagement with mobile health-based exercise interventions, including limited health literacy, concerns about operational complexity, uncertainty about exercise safety, difficulty maintaining motivation, and competing daily priorities. These findings indicated that the intervention should not rely solely on the digital delivery of exercise content. Instead, participants required a program that combined professional guidance, simplified digital operation, personalized health knowledge, progressive exercise lessons, and repeated motivational support.

Three development-relevant needs were particularly important. First, participants emphasized the need for professional engagement to increase trust, perceived safety, and confidence in exercise participation. This finding informed the inclusion of technical guidance, professional support, exercise video feedback, and expert-authority framing reminders. Second, participants preferred personalized, easily understandable health information over abstract health education. This finding informed the use of simplified health education materials, illustrated content, tailored knowledge delivery, and feedback-based psychological adaptation. Third, participants preferred exercise lessons that were beneficial, tailored, dynamic, fragmented, and moderately challenging. This finding informed the design of personalized multicomponent exercise lessons, goal setting, self-monitoring, and progressive motivational support. Participants also expressed positive attitudes toward several nudge strategies, including simplification, reminders, feedback, social norms, expert authority, gamification, economic framing, and precommitment. These findings were used, together with the scoping review findings, to inform the theory-informed development of the preliminary intervention program.

### 3.3 Development of the preliminary intervention program

Based on the integrated mapping of scoping review findings and qualitative interview findings, a preliminary intervention program was developed. Evidence from the scoping review, user needs and preferences from the qualitative interviews, Capability, Opportunity, Motivation–Behavior domains, selected nudge strategies, resulting intervention components, and the logic for matching were integrated in a mapping table (Supplementary Table 5). This mapping was used to make the development logic explicit and to support the translation of evidence and user needs into concrete intervention components. The preliminary intervention program derived from this process is shown in Supplementary Table 6.

### 3.4 Expert consultation and refinement

#### 3.4.1 Demographics of the expert consultation

A total of 12 experts participated in the structured expert consultation, including eight specialists in clinical medicine or nursing, two in rehabilitation medicine, and two in software development. The expert group provided clinical, nursing, rehabilitation, and technical perspectives on refining the preliminary intervention program. Detailed expert characteristics are shown in Supplementary Table 7.

#### 3.4.2 Expert feedback on the preliminary intervention program

Experts provided seven categories of recommendations. These focused on: (1) adjusting the timing and setting of the intervention; (2) clarifying the distinction between exercise prescriptions and behavioral support strategies; (3) refining the Capability, Opportunity, Motivation–Behavior domains and related intervention formats; (4) adding psychological adaptation support; (5) strengthening professional support; (6) revising health education materials; and (7) optimizing selected nudge strategies. Detailed expert comments are summarized in Supplementary Table 8. These recommendations led to several revisions. The intervention was reorganized into progressive stages of delivery to support the transition from supervised community exercise to home-based exercise. Psychological adaptation was added to address fear of exercise, low confidence, frustration with technology, and competing daily priorities. Professional support was refined to include real-time consultation, monthly progress review, weekly video feedback, and manual follow-up when needed. Health education materials were revised to improve clarity, personalization, and age-friendliness. Selected nudge strategies were further aligned with frail older adults’ needs, behavioral barriers, and home-based delivery contexts. The revised program was returned to the experts to check whether their comments had been accurately understood and incorporated.

### 3.5 Final intervention program and digital translation for delivery

Following structured expert consultation, the research team incorporated the experts’ recommendations and finalized the intervention program. The final program consisted of 13 intervention components targeting capability, opportunity, and motivation. After finalization, the program was translated into digital delivery for future implementation. The theoretical mapping of behavioral targets, component types, nudge strategies, proposed theory-informed mechanisms, and intervention components is presented in Table 1. The operational content, timing, and delivery channels of each intervention component are presented in Table 2, and detailed examples of framing reminders and gamification rules are provided in Table 3.

**Table 1:** Mapping of Capability, Opportunity, Motivation–Behavior domains, component types, nudging strategies, proposed theory-informed mechanisms, and intervention components.

Capability, Opportunity, Motivation–Behavior domain	Component type	Main nudging strategies	Proposed theory-informed mechanism	Intervention component
Physical capability	Core	Priming; information support; simplification	Reduce information-seeking costs and operational burden, minimize decision fatigue, and increase familiarity with the home-based exercise system.	1.1 Technical guidance
Physical capability	Core	Information support; default options; simplification; social norms	Reduce information neglect and bounded attention by presenting exercise-related information in concrete, salient, and easy-to-understand forms.	1.2 Health education
Physical capability	Core	Information support; simplification; default options	Reduce choice overload and cognitive effort by providing default, structured, and personalized multicomponent exercise options.	1.3 Exercise lessons
Psychological capability	Supportive/adaptable	Emotional anchoring	Use emotional anchoring and affective imagery to make future health consequences more concrete and personally relevant.	1.4 Future self-visualization
Psychological capability	Supportive/adaptable	Feedback	Strengthen perceived control and confidence by providing feedback on barriers, progress, and adaptive coping.	1.5 Psychological adaptation
Physical opportunity	Supportive/adaptable	Environmental cues	Use contextual and spatial cues to activate exercise-related intentions and make home-based exercise more visible and accessible.	2.1 Environmental modification
Social opportunity	Core	Feedback; expert authority	Leverage expert authority and trust heuristics to increase confidence, perceived safety, and acceptance of exercise recommendations.	2.2 Professional support
Social opportunity	Supportive/adaptable	Social norms; feedback	Use social norms and feedback to increase perceived normality, mutual encouragement, and social reinforcement of exercise participation.	2.3 Peer support
Reflective motivation	Core	Feedback; environmental cues	Provide visible feedback on exercise completion and progress to strengthen self-regulation and immediate reinforcement.	3.1 Self-monitoring
Reflective motivation	Core	Precommitment; default options	Use precommitment and default goals to reduce time inconsistency and support translation of intention into repeated action.	3.2 Goal setting
Automatic motivation	Core	Framing; reminders; social norms; peer comparison; expert authority	Use reminders and gain, loss, social, expert, and personalized framing to increase salience, reduce planning neglect, and activate motivational responses.	3.3 Framing reminders
Reflective motivation	Supportive/adaptable	Information support; social norms; peer comparison	Use social norms, peer comparison, and informational support to enhance perceived achievability and motivation through similar others’ examples.	3.4 Role modeling
Automatic motivation	Supportive/adaptable	Gamification; peer comparison	Use immediate rewards, point deduction, and peer comparison to activate achievement gratification, loss aversion, and sustained engagement.	3.5 Gamification design

Note. The proposed mechanisms were derived from nudge theory, the dual-process perspective, and prior literature; they were not empirically tested in the present intervention development study. Core components refer to intervention elements intended to be delivered to all participants because they are central to safe exercise delivery, mobile health use, and repeated engagement. Supportive/adaptable components refer to elements that can be tailored according to participants' needs, digital literacy, social preferences, and implementation context. Components numbered 1, 2, and 3 correspond to capability-, opportunity-, and motivation-related targets, respectively. The detailed operational content of each intervention component is presented in Table 2.

**Table 2:** Operational content of the final mobile health-based nudging intervention program.

Intervention component	Operational content	Timing and platform
1.1 Technical guidance	1) Face-to-face instruction on home-based exercise system usage and precautions. 2) Provide illustrated user manuals with supplementary videos and minimizing text. 3) Technical support via WeChat group.	Weeks 1–2 face-to-face instruction; weeks 3–4 question-and-answer support; weeks 4–12 remote instruction; Offline; WeChat
1.2 Health education	1) Health education covers five domains: frailty improvement, appropriate exercise, chronic disease management, digital literacy enhancement, and psychological adaptation, with automatic subscription and push notifications. 2) Enhanced information salience through using illustrations. 3) Incorporated peer examples to leverage the demonstration effect, utilizing social identity to reduce psychological resistance. 4) Information presentation techniques: replaced abstract concepts with concrete actions; bundled health education with daily habits; materialized health risks.	Automatic push notifications at 10:00 AM every Monday, Wednesday, and Friday for 12 weeks; Home-based exercise system
1.3 Exercise lessons	1) Personalized Chinese version VIVIFRAIL multicomponent exercise videos based on exercise prescriptions. 2) Two versions for each level of difficulty: the instructor's version contains professional instruction, including explanation of movements, points and notes. The self-exercise version has no narration and is rhythmically based with background music to put participants in the mood for the exercise.	Stage-specific timing. Automatic push notifications at 10:00 AM every Monday, Wednesday, and Friday for 12 weeks in home-based exercise system
1.4 Future self-visualization	Guide participants to visualize the contrast between their future self with consistent exercise and without exercise, using concrete imagery of future health states to evoke emotional engagement.	Monthly; Home-based exercise system
1.5 Psychological adaptation	Provide regular psychological support to tap into the barriers of exercise participation and increase confidence.	Monthly; Home-based exercise system
2.1 Environmental modification	Provide suggestions for home exercise environments, such as prominently displaying exercise equipment and “Exercise Every Day” reminder signs.	Initial setup and ongoing use; Offline
2.2 Professional support	1) Provides online consultation services by professionals to address exercise-related queries in real-time. 2) Monthly guided self-assessment of physical changes (e.g., “Compared to before, can you now climb 10 steps more easily?”), with positive reinforcement of exercise adherence. 3) Participants are encouraged to submit weekly exercise videos with movement corrections and personalized feedback from professionals.	Continuous real-time consultation; monthly progress review; weekly video feedback; Home-based exercise system
2.3 Peer support	1) Encourage participants to share experiences and interactions in virtual communities. 2) Provides structured moderator reinforcement (e.g., affirming comments on progress posts).	Ongoing/as needed; Home-based exercise system
3.1 Self-monitoring	1) Distribute a printed exercise behaviour log calendar and hang it in a prominent location at home. Participants are required to place a star sticker on the calendar when they complete a scheduled exercise session. 2) Home-based exercise system automatically monitors and visually displays the exercise duration and health status, using progress bars to show the completion rate of the exercise plan.	Real-time updates; Offline; home-based exercise system
3.2 Goal setting	1) Set goals with older adults and encourage them to make a written precommitment: complete health education learning and multicomponent exercise three times a week. 2) Review the implementation of the commitment at the stage and adjust the goals when appropriate.	Monthly; Offline; home-based exercise system
3.3 Framing reminders	Nine types of framing reminders were designed, including gain framing, loss framing, economic benefits framing, long-term benefits framing, healthy age framing, time-perception framing, expert authority framing, peer comparison framing, and personalized framing. Detailed examples are provided in Table 3.	Automatic reminders sent at 10:00 AM every Monday, Wednesday, and Friday. If no response, a second reminder follows at 5:00 PM the same day. If still no response, a manual follow-up is conducted; Home-based exercise system
3.4 Role modeling	1) Regularly publish success stories in the WeChat group chat to utilize role modeling to support motivation. 2) Recognize the top scorers and the exercise star of the month in the WeChat group chat, using social norms and peer comparison to motivate participation.	Monthly; WeChat
3.5 Gamification design	Points ranking, Exercise Star, and reward mechanisms were designed to support participation and sustained engagement. Detailed rules are provided in Table 3.	Real-time; Home-based exercise system

Note. The behavioral targets, nudging strategies, and proposed theory-informed mechanisms corresponding to each intervention component are summarized in Table 1. Detailed examples of framing reminders and gamification rules are shown separately in Table 3 to improve readability while preserving operational detail.

**Table 3:** Examples of framing reminders and gamification rules.

Category	Type / element	Operational description
Framing reminder	Gain framing reminder	Emphasize the benefits of exercise. “Keeping active can slow down aging and keep you young and energetic, come join us!” Or “Exercise not only improves your physical fitness, it also improves your mood, enjoy a relaxing moment together.”
	Loss framing reminder	Emphasize the potential negative effects of physical inactivity. “Lack of exercise can lead to osteoporosis and increase the risk of cardiovascular disease, so get moving.”
	Economic benefits framing reminder	Emphasize that regular exercise can reduce medical costs and family financial burdens.
	Long-term benefits framing reminder	Emphasize the 3-month, 6-month, and 1-year benefits of regular exercise.
	Healthy age framing reminder	Relate the effects of exercise to biological age. “Regular exercise can make your healthy age 5 years younger than your actual age.”
	Time-perception framing reminder	Compare the time spent exercising to daily activities. “Spending 30 min a day exercising is equivalent to watching only one episode of TV.”
	Expert authority framing reminder	Use advice from experts and authorities to increase credibility. “National physical activity guidelines recommend regular moderate-intensity exercise. Have you exercised today?”
	Peer comparison framing reminder	Stimulate intrinsic competitiveness by comparing peer exercise data. “80% of seniors completed 3 exercise sessions this week.”
	Personalized framing reminder	Tailor exercise recommendations to an individual’s health status, preferences, and goals.
Gamification rule	Points ranking	Ranking based on points; initial points are 100 points; 10 points are deducted for not completing an exercise, triggering a framing reminder.
	Exercise Star	The title of “Exercise Star” is awarded to participants who have recorded the longest exercise duration.
	Reward	Participants who have 100 points at the end of the intervention or who have been awarded the title of “Exercise Star” can receive a small gift.

Note. The examples are illustrative and can be tailored according to participants' health status, preferences, and exercise progress.

#### 3.5.1 Intervention timing and setting

The finalized mobile health-based nudging intervention was structured into three progressive stages of delivery. During the initial adaptation stage, participants would attend three supervised group exercise sessions per week at community centers in Weeks 1–2. These sessions would provide in-person exercise instruction and training in using the home-based exercise system, helping participants build foundational skills and confidence. During the transition stage in Weeks 3–4, a hybrid approach would be adopted, consisting of one weekly supervised community-based session and two weekly home-based sessions delivered through the exercise system, to support a gradual transition from supervised to independent exercise. During the consolidation stage in Weeks 5–12, the intervention would transition to home-based delivery, with three exercise sessions per week supported by the mobile health platform and embedded nudge strategies.

To reduce operational barriers and potential digital exclusion during the transition, technical guidance would be provided through face-to-face demonstrations, illustrated manuals, short videos, WeChat-based question-and-answer support, remote troubleshooting, and manual follow-up when participants did not respond to system prompts. Psychological adaptation support would be provided through monthly check-ins to identify barriers such as fear of exercise, low confidence, frustration with technology, family-related constraints, or competing daily priorities, followed by encouragement, problem-solving support, and adjustment of reminders when necessary.

#### 3.5.2 Intervention delivery channels and digital translation

The mobile health-based nudging intervention was designed to be delivered through three complementary channels: face-to-face support, WeChat, and the YaMove WeChat Mini Program. The intervention delivery structure and functional modules of the digital platform are shown in Fig. 3. The YaMove WeChat Mini Program would be used to deliver health education, exercise lesson recommendations, notification alerts, exercise logging, professional feedback, virtual community functions, and incentive rewards. WeChat-based support would be used for professional communication, role modeling, peer interaction, psychological support, and technical question-and-answer support. Offline support would be retained for initial technical guidance, supervised exercise instruction, environmental modification, and paper-based self-monitoring. The digital platform was designed to be simple, efficient, and age-friendly, with streamlined procedures and minimal operational complexity to facilitate use among community-dwelling frail older adults.

**Fig. 3:**
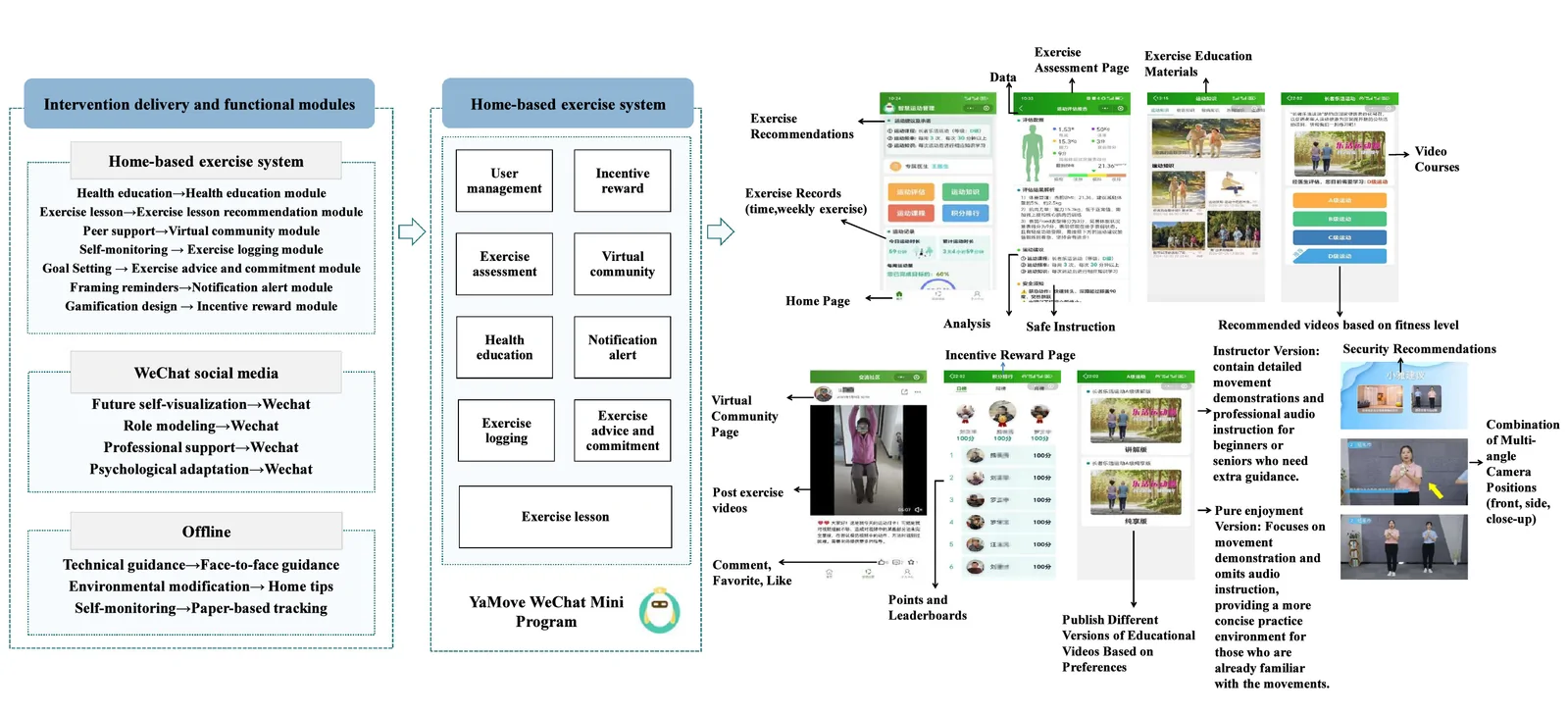
Intervention delivery channels, functional modules, and representative digital interfaces. Note: The intervention was designed to be delivered through the YaMove WeChat Mini Program, WeChat-based support, and offline support. Representative screenshots illustrate the planned digital delivery functions and do not represent usability, acceptability, or effectiveness evaluation. Chinese screenshots are used as representative interfaces, with key planned functions annotated in English.

#### 3.5.3 Content of the final intervention program

The final mobile health-based nudging intervention was grounded in the Capability, Opportunity, Motivation–Behavior model and addressed three core behavioral domains: capability, opportunity, and motivation. Core components included technical guidance, health education, exercise lessons, professional support, self-monitoring, goal setting, and framing reminders. These components were intended to be delivered to all participants because they were central to safe exercise delivery, mobile health use, and repeated engagement. Supportive/adaptable components included future self-visualization, psychological adaptation, environmental modification, peer support, role modeling, and gamification design. These components were designed to enhance engagement and could be adapted according to participants’ needs, digital literacy, social preferences, and local implementation resources. The 14 nudge strategies were not intended to function as independent experimental components in this development stage; rather, each strategy was selected to address a specific behavioral barrier identified through the scoping review, qualitative interviews, or expert consultation. Together, these approaches may facilitate exercise-related decision-making and support exercise behavior among frail older adults (Table 1).

## 4 Discussion

This study developed a 12-week nurse-led mobile health-based nudging intervention program for community-dwelling frail older adults. The program was developed through evidence synthesis, qualitative interviews, theory-informed mapping, structured expert consultation, and digital translation for delivery. It specifies behavioral targets, nudge strategies, intervention components, delivery procedures, and platform-based delivery functions. Importantly, this study should be interpreted as an intervention development study rather than a feasibility, pilot, or effectiveness study. Future studies are needed to empirically evaluate the feasibility, acceptability, safety, implementation procedures, and effectiveness of the developed program.

Previous exercise promotion interventions for older adults have often followed the Behavior Change Wheel pathway, in which determinants identified by the Capability, Opportunity, Motivation–Behavior model are translated into intervention functions and specific Behavior Change Techniques (Chen et al., 2023; Kwok et al., 2021). In the present study, the Capability, Opportunity, Motivation–Behavior model was used to identify whether exercise-related barriers were mainly located in capability, opportunity, or motivation, whereas nudging was used to redesign the everyday decision environment in which home-based exercise occurs. This choice reflects the aim of developing a low-burden, acceptable, and daily-life-embedded mobile health intervention prototype for frail older adults. For this population, non-adherence may arise not only from insufficient knowledge or intention, but also from high perceived effort, low confidence, limited self-regulatory capacity, and digital operation burden. A taxonomy of Behavior Change Techniques is useful for naming and classifying intervention content, whereas nudging directs attention to how support is placed in the environment so that exercise becomes easier to start and maintain without restricting autonomy (Dolan et al., 2012; Thaler and Sunstein, 2008).

Although mobile health can extend exercise support from community settings into the home, digital delivery alone may also create participation barriers for frail older adults, particularly when digital literacy, confidence, or technical support is limited (Solis-Navarro et al., 2022; Spann and Stewart, 2018; Stookey and Katzel, 2020; The Lancet Digital Health, 2023). The present program therefore does not treat technology as a substitute for human support. Instead, its hybrid design combines platform-based support with initial guidance and ongoing nurse communication, which may help balance the scalability of digital delivery with the reassurance and support needed by frail older adults (Kebede et al., 2022; Peng et al., 2024). Future pilot testing should examine not only whether participants use the platform, but also which digital functions are difficult to use, how often technical assistance is required, and whether technical burden differs by age, education, living arrangement, sensory function, or previous digital experience.

The exercise component of the program draws on multicomponent exercise recommendations and VIVIFRAIL-related evidence for frailty management, while its delivery structure adapts these elements to a nurse-led, mobile health-based, community-oriented context (Dent et al., 2019; World Health Organization, 2019). In routine community nursing practice, this program could be used as a staged support pathway for frail older adults, but its delivery details should be further refined through pilot testing. Future pilot work should examine recruitment, retention, usability, acceptability, adherence, preliminary safety, implementation procedures, and how participants experience different nudging strategies in daily life. It should also assess whether the program remains flexible enough for participants to adjust goals or reminder frequency when needed, and what level of community nurse support is appropriate for maintaining engagement without increasing unnecessary workload. These findings will help optimize the intervention while preserving its low-burden and community-oriented design. A randomized controlled trial will then be needed to evaluate effects on exercise behavior, frailty-related outcomes, and sustained participation.

## 5 Limitations

Several limitations should be acknowledged. First, this was an intervention development study; feasibility, usability, acceptability, safety, implementation procedures, and effectiveness were not empirically tested. Second, expert consultation was conducted via a structured synchronous meeting rather than through a Delphi process or a quantitative content-validity assessment. No formal voting, Likert-scale ratings, consensus rate, or content validity index was calculated, limiting the quantification of expert agreement. Third, the expert panel did not include community caregivers or social workers, which may have limited input on home environmental modification, community resource coordination, and long-term implementation in real-world community settings. Fourth, the study was conducted in a single Chinese community context, and cultural adaptability to other healthcare systems requires further evaluation. Finally, because multiple nudge strategies were incorporated simultaneously, the relative contribution and optimal combination of individual strategies remain unclear.

## 6 Conclusions

This study developed a nurse-led mobile health-based nudging intervention program for community-dwelling frail older adults through scoping review, qualitative interviews, theory-informed mapping, and structured expert consultation. The program specifies behavioral targets, nudge strategies, intervention components, delivery procedures, and digital translation for future implementation. As this study focused on intervention development rather than validation, future studies are needed to evaluate its feasibility, acceptability, safety, implementation procedures, and effectiveness.

## Ethics approval statement

Our study was approved by the College Ethics Review Committee of Xiangya Nursing School of Central South University (Approval Number: E202463).

## Funding statements

This study was supported by grants from the Natural Science Foundation of Hunan Province (2026JJ60564).

## Patient consent statement

All participants, both patients and specialists, signed an informed consent form.

## CRediT authorship contribution statement

**Ruotong Peng:** Writing – review & editing, Writing – original draft, Visualization, Validation, Supervision, Software, Resources, Project administration, Methodology, Investigation, Formal analysis, Data curation, Conceptualization. **Jia Yu:** Formal analysis, Data curation, Conceptualization. **Ronghua Yang:** Formal analysis, Data curation, Conceptualization. **Hui Feng:** Writing – review & editing, Writing – original draft, Funding acquisition, Data curation, Conceptualization. **Yinan Zhao:** Writing – review & editing, Writing – original draft, Funding acquisition, Data curation, Conceptualization.

## Declaration of competing interest

The authors declare that they have no known competing financial interests or personal relationships that could have appeared to influence the work reported in this paper.

## Data Availability

The authors made the raw data supporting this article's conclusion available without undue reservation.
