# Supplementary material for: Nurse-led mobile health-based nudging intervention program to support exercise behavior among community-dwelling frail older adults: A multiphase intervention development study
Source: Int J Nurs Stud Adv. 2026 Sep 24;11:100690. doi: 10.1016/j.ijnsa.2026.100690 (PMC13672859; doi:10.1016/j.ijnsa.2026.100690)
Supplement: Supplementary file 1 [file mmc1.docx]

**Supplementary files**

Supplementary Table 1. Completed GUIDED reporting checklist

Supplementary Table 2. Search syntax

Supplementary Table 3. Interview Guidance

Supplementary Table 4. The characteristics of the included study

Supplementary Table 5. Integrated mapping of evidence, user needs, nudging strategies, and intervention components

Supplementary Table 6. The preliminary intervention program

Supplementary Table 7. Experts' demographic characteristics

Supplementary Table 8. Expert suggestions and revisions

Supplementary Figure 1. The PRISMA flow diagram

## Supplementary Table 1. Completed GUIDED reporting checklist

The present intervention development study was reported using the Guidance for Reporting Intervention Development Studies in Health Research (GUIDED) checklist. The wording below is adapted for the present manuscript and indicates where each reporting item is addressed.

| **Item** | **GUIDED reporting item** | **Where addressed in this manuscript** | **Authors' notes** |
| --- | --- | --- | --- |
| 1 | Context of intervention development | Introduction; Methods 2.1; Methods 2.4; Discussion | The manuscript describes the intervention context as community-based frailty management in China, the low participation and adherence to exercise among frail older adults, and the need for an accessible mobile health-supported exercise program. |
| 2 | Purpose of the intervention development process | Abstract; Aims; Methods 2.1 | The purpose was to develop a nurse-led mobile health-based nudging intervention program to support exercise behavior among community-dwelling frail older adults. |
| 3 | Target population for the intervention | Abstract; Introduction; Aims; Methods 2.4; Results 3.2; Table 1 | The target population is community-dwelling older adults with frailty or pre-frailty. The qualitative phase included older adults with pre-frailty or frailty to inform the identification of needs and preferences. |
| 4 | Contribution of any published intervention development approach | Methods 2.1: Strengths and limitations | The study used a pragmatic, multiphase intervention development design and was reported in accordance with the GUIDED checklist. A full formal intervention development framework, such as the Medical Research Council framework or Intervention Mapping, was not fully implemented; this is clarified to avoid overstatement. |
| 5 | Evidence sources used to inform intervention development | Methods 2.3-2.5; Results 3.1-3.4; Supplementary Tables 1-8 | The intervention was informed by a scoping review, semi-structured qualitative interviews with frail older adults, and multidisciplinary expert consultation. These sources informed the preliminary program and subsequent refinements. |
| 6 | Use of published theory | Methods 2.2; Results 3.3; Results 3.5; Discussion; Figure 2; Figure 3; Supplementary Table 5 | The Capability, Opportunity, Motivation-Behavior model was used to identify behavioral determinants, while nudge strategies were used to translate these determinants into practical intervention components in a mobile health context. |
| 7 | Use or adaptation of components from existing interventions | Methods 2.3; Results 3.1; Results 3.5; Table 1; Supplementary Tables 3 and 4 | The intervention incorporated and adapted components from previous mobile health exercise interventions, including technical support, health education, reminders, feedback, goal setting, social support, and multicomponent exercise lessons based on the Chinese version of VIVIFRAIL. |
| 8 | Guiding principles, people, or factors prioritized during development | Methods 2.2; Methods 2.4; Methods 2.5; Results 3.2; Results 3.5; Table 1; Discussion | Key priorities included age-friendly design, low burden, safety, preservation of autonomy, professional support, accessibility for home-based exercise, acceptability among frail older adults, and feasibility of nurse-led implementation. |
| 9 | Stakeholder contribution to the development process | Methods 2.4; Methods 2.5; Results 3.2; Results 3.4; Supplementary Tables 2, 7, and 8 | Community-dwelling frail older adults contributed through qualitative interviews. Experts from clinical medicine, nursing, rehabilitation medicine, and software development contributed through expert consultation and confirmation of the revised program. |
| 10 | Changes in intervention content or format during development | Results 3.3; Results 3.4; Results 3.5; Table 1; Supplementary Tables 6 and 8 | The preliminary program was revised after expert consultation. Revisions included adjustments to intervention timing and setting, a distinction between exercise prescription and behavioral strategies, the addition of psychological support, the refinement of professional support, the revision of health education content, and the optimization of nudge strategies. |
| 11 | Changes required or likely to be required for subgroups | Results 3.5; Table 1; Discussion; Future directions | The program includes tailoring through individualized exercise prescriptions and technical support. Future adaptations may be needed according to frailty severity, exercise capacity, chronic disease status, digital literacy, cognitive status, and family or community support. |
| 12 | Important uncertainties remaining after development | Strengths and limitations; Future directions; Conclusions | Feasibility, usability, and effectiveness testing were not conducted in this development study. Remaining uncertainties include intervention feasibility, user acceptability, effectiveness, implementation burden, optimal combinations of nudge strategies, economic feasibility, scalability, and cross-cultural transferability. |
| 13 | Description of the developed intervention using intervention reporting principles | Results 3.5; Tables 1–3; Figure 3 | The developed intervention is described in terms of components, behavioral targets, nudge strategies, intervention content, delivery timeline, and platform. These details correspond to the key Template for Intervention Description and Replication reporting domains, although a separate full checklist was not completed in this manuscript. |
| 14 | Availability of the intervention development report | Manuscript and supplementary files; Data availability statement | The intervention development process is reported in the manuscript and supplementary files. If accepted, the article and supplementary materials will be available according to the journal's publication policy. Additional data may be requested from the corresponding author where appropriate. |

Reference: Duncan, E., O'Cathain, A., Rousseau, N., Croot, L., Sworn, K., Turner, K. M., Yardley, L., & Hoddinott, P. (2020). Guidance for reporting intervention development studies in health research (GUIDED): an evidence-based consensus study. BMJ Open, 10, e033516. https://doi.org/10.1136/bmjopen-2019-033516

## Supplementary Table 2. Search syntax

| PubMed (N=323) | ("digitalisation"[All Fields] OR "digitalised"[All Fields] OR "digitalization"[All Fields] OR "digitalize"[All Fields] OR "digitalized"[All Fields] OR "digitalizer"[All Fields] OR "digitalizing"[All Fields] OR "digitally"[All Fields] OR "digitals"[All Fields] OR "digitization"[All Fields] OR "digitizations"[All Fields] OR "digitize"[All Fields] OR "digitized"[All Fields] OR "digitizer"[All Fields] OR "digitizers"[All Fields] OR "digitizes"[All Fields] OR "digitizing"[All Fields] OR "radiographic image enhancement"[MeSH Terms] OR ("radiographic"[All Fields] AND "image"[All Fields] AND "enhancement"[All Fields]) OR "radiographic image enhancement"[All Fields] OR "digital"[All Fields] OR ("internet"[MeSH Terms] OR "internet"[All Fields] OR "internet s"[All Fields] OR "internets"[All Fields]) OR "web"[All Fields] OR ("mobile"[All Fields] OR "mobiles"[All Fields]) OR "online"[All Fields]) AND ("exercise"[MeSH Terms] OR "exercise"[All Fields] OR "exercises"[All Fields] OR "exercise therapy"[MeSH Terms] OR ("exercise"[All Fields] AND "therapy"[All Fields]) OR "exercise therapy"[All Fields] OR "exercising"[All Fields] OR "exercise s"[All Fields] OR "exercised"[All Fields] OR "exerciser"[All Fields] OR "exercisers"[All Fields] OR "physical activity"[All Fields] OR "physical activities"[All Fields] OR "exercise program"[All Fields] OR "exercise class"[All Fields] OR "exercise training"[All Fields]) AND ("older adults"[All Fields] OR "older people"[All Fields] OR ("elder s"[All Fields] OR "elders"[All Fields] OR "sambucus"[MeSH Terms] OR "sambucus"[All Fields] OR "elder"[All Fields]) OR ("aged"[MeSH Terms] OR "aged"[All Fields] OR "elderly"[All Fields] OR "elderlies"[All Fields] OR "elderly s"[All Fields] OR "elderlys"[All Fields]) OR "older population"[All Fields] OR "senior citizen"[All Fields]) AND ("frail"[All Fields] OR "frails"[All Fields] OR "frailty"[MeSH Terms] OR "frailty"[All Fields] OR "frailness"[All Fields] OR ("frailty"[MeSH Terms] OR "frailty"[All Fields] OR "frailties"[All Fields]) OR "pre-frailty"[All Fields]) |
| --- | --- |
| Web of Science (N=520) | TS=((digital OR internet OR web OR mobile OR online) AND (exercise OR "physical activity" OR "physical activities" OR "exercise program" OR "exercise class" OR "exercise training") AND ("older adults" OR "older people" OR elder OR elderly OR "older population" OR "senior citizen") AND (frail OR frailty OR pre-frailty)) |
| Embase (N=729) | (digital OR 'internet'/exp OR internet OR 'web'/exp OR web OR mobile OR 'online'/exp OR online) AND ('exercise'/exp OR exercise OR 'physical activity'/exp OR 'physical activity' OR 'physical activities' OR 'exercise program'/exp OR 'exercise program' OR 'exercise class' OR 'exercise training'/exp OR 'exercise training') AND ('older adults'/exp OR 'older adults' OR 'older people'/exp OR 'older people' OR elder OR 'elderly'/exp OR elderly OR 'older population' OR 'senior citizen'/exp OR 'senior citizen') AND (frail OR 'frailty'/exp OR frailty OR 'pre frailty'/exp OR 'pre frailty') |
| CINAHL (N=100) | (digital or internet or web or mobile, or online) AND (exercise or "physical activity" or "physical activities" or "exercise program" or "exercise class" or "exercise training") AND ("older adults" or "older people" or elder or elderly or "older population" or "senior citizen") AND (frail or frailty or pre-frailty) |
| Cochrane (N=579) | ((digital or internet or web or mobile or online) AND (exercise or "physical activity" or "physical activities" or "exercise program" or "exercise class" or "exercise training") AND ("older adults" or "older people" or elder or elderly or "older population" or "senior citizen") AND (frail or frailty or pre-frailty)) |

## Supplementary Table 3. Interview Guidance

| **Dimension** | | **Interview Questions** |
| --- | --- | --- |
| Previous Experience | | Have you ever exercised at home by watching exercise videos, using exercise apps, or fitness websites? What are your thoughts? Would you be willing to try it if you haven't tried it?  *(Note: For experienced users, we will evaluate their preferred mobile exercise apps. For those new to such apps, we can refer to apps like "Keep" as examples.)* |
| Willingness to Use | | What factors influence your decision to exercise using exercise apps?  *Note: Time and effort, length/timing of the course, health status, authoritative recommendations, etc.* |
| Technical Assistance | | What guidance and training do you expect before using exercise apps? What kind of assistance would you like during the usage process? |
| Interface Design | | Describe your expectations and preferences for exercise apps.  *Note: Inquire about colors, fonts, icons, layouts (multifunctional options/show key features only), etc., and if necessary, create from the pen and paper provided to seniors.* |
| Requirements | Modules | What modules should exercise apps for older adults include? |
|  | Guidance | What exercise, educational resources, and counseling would you like to receive? |
|  | Data | Which health and exercise data interests you, and how would you prefer it presented? |
| Nudges | Reminder | What type of reminders would motivate you to use exercise apps, and how frequently would you like to be reminded? |
|  | Goal-setting | Have you set exercise goals in the past? If not, what would be the reason? |
|  | Social influence | Would you like to share your exercise progress and communicate with others? Why or why not? |
|  | Gamification | What do you view as gamification design elements (e.g., challenges, levels, points, and rewards)? Do these elements entice you to use mobile exercise apps? |
|  | Social competition | How would you feel if your friends, family, or peers were using the exercise apps and making more progress than you? |
|  | Precommitment | We may ask you to sign a commitment before using the exercise app. What are your thoughts on this? |
|  | Privacy and security | What concerns or worries do you have about used exercise apps? |

## Supplementary Table 4. The characteristics of the included study

| **Author/Year** | **Country** | **Study design** | **Intervention method** | **Exercise type** | **Behavioral intervention strategies** | **Applied theories** | **Outcome measures** |
| --- | --- | --- | --- | --- | --- | --- | --- |
| Zhang Jiajia, 2023 [1] | China | Thesis | "YiDong" WeChat Mini Program | Multicomponent Exercise | Self-monitoring, goal setting, feedback, reminders, professional guidance, progress tracking, and visualization. | Technology Acceptance Model, Health Belief Model | General information questionnaire, frailty scale, 6-minute walk test, adherence & usability scale |
| Zhu Yan, 2021 [2] | China | Thesis | Sports and health management platform | Resistance training | Education, social support, and feedback | Health Belief Model | Physiological outcome, frailty outcomes, and psychological & social outcomes |
| Weiss et al., 2023 [3] | Canada | Cross-sectional study | Zoom or Microsoft Teams | Strength and balance training | Health education | / | Demographics, care delivery variables, cognitive scale, frailty scale, fall-related metrics, and balance measurements. |
| Zhang et al., 2022 [4] | China | RCT protocol | Gait monitoring app and smart insoles | 30 types of exercise | Reminders, monitoring, and feedback | / | Physical function, gait parameters, frailty score, weekly MVPA, psychological and cognitive measures, adherence, and safety |
| Meredith et al., 2023 [5] | UK | Quasi-experimental protocol | Zoom | Resistance training | Open-ended questions, active listening, reflective practice, goal setting, peer support, technical assistance, and digital literacy improvement | / | Recruitment rate, training rate, retention rate, participation rate, adherence rate, acceptability interviews, physical function, appetite, well-being, QoL, anxiety, depression, self-management efficacy, chronic diseases, physical activity |
| Kerstiens et al., 2023 [6] | USA | Design science research | BeFitMe™ + Apple Watch + Zoom | Progressive lower-body strength, flexibility, and walking training | Behavior change text messages (reminders, suggestions, feedback, motivation) | Behavior change wheel | Identifying and overcoming barriers to the intervention process, usability (% preferring to wear and use, % adhering to physical activity, % notifying of response, % of days data generated, % of days wearing the watch) |
| Villa-García et al., 2023 [7] | Spain | Co-design study | ViviFrail APP | Multicomponent exercise | 27 strategies categorized into: reducing barriers, promoting app use, encouraging virtual group participation, external support (prioritized: digital literacy, competency assessment, home tech support, weekly professional calls, personalized & group virtual sessions) | / | Implementation strategies and evaluation priorities |
| Geraedts et al., 2017 [8] | Netherlands | Feasibility study | Sensors and app | Otago Kitchen Table Exercise (multicomponent) | Motivational support, problem-solving, personalized guidance, visual cues, real-time feedback, data visualization, goal setting, and self-efficacy | / | Exercise adherence (% completing 5 sessions/week), sensor wear (≥70% = adequate adherence), reported issues, user feedback questionnaire. |
| Geraedts et al., 2021 [9] | Netherlands | Pilot study | Sensors and app | Otago Kitchen Table Exercise (multicomponent) | Personalized guidance, real-time feedback, visual cues, progressive difficulty | Transtheoretical Model, Social Cognitive Theory | Demographics, physical function (sit-to-stand test), daily physical activity (SQUASH questionnaire) |
| Hong and Kong, 2023 [10] | South Korea | RCT | VR platform | Resistance training | Supervision | / | Body composition, physical function, and frailty index |
| Okpara et al., 2023 [11] | Canada | Feasibility study | Zoom | Multicomponent exercise | / | / | Feasibility: reach, effectiveness, adoption, implementation, maintenance |
| Wang et al., 2022 [12] | Canada | Feasibility study | Phone | Multicomponent exercise | / | / | Recruitment rate, retention rate, adherence rate, physical function, physical activity, dietary intake, self-efficacy, fatigue, self-rated health, nutritional risk, mental health, QoL, adverse events, participant feedback |
| Zhu et al., 2021 [13] | China | RCT | Online-offline hybrid | Multicomponent exercise | Education, social support, peer support, supervision, and feedback | / | Physical function, body composition, psychological and social support, covariates, adherence & adverse events |
| Yoo et al., 2023 [14] | South Korea | Quasi-experimental study | Tablet and VR glasses | Multicomponent exercise | / | / | User experience questionnaire |

Abbreviations: RCT: Randomized Controlled Trial ; MVPA: Moderate-to-Vigorous Physical Activity ; QoL: Quality of Life ; APP: Application ; VR: Virtual Reality; SQUASH: Short Questionnaire to Assess Health-Enhancing Physical Activity

**References**

1.	张佳佳. 基于移动医疗的社区衰弱老年人居家运动康复平台的构建 [D]: 郑州大学; 2023.

2.	朱岩. 基于“互联网+”的社区衰弱及衰弱前期老年人运动干预方案的构建与应用 [D]: 蚌埠医科大学; 2021.

3.	Weiss SM, Castelo M, Liu B, Norris M. Virtual fall program assessment for frail Canadian community-dwelling older adults: Examining equitable accessibility. Digit Health. 2023 Jan-Dec;9:20552076231178410.

4.	Zhang X, Li J, Sui X, Xu L, Zhu L, Pang Y, et al. Effects of Remotely Supervised Physical Activity on Health Profile in Frail Older Adults: A Randomized Controlled Trial Protocol. Front Aging Neurosci. 2022;14:807082.

5.	Meredith SJ, Roberts H, Grocott MPW, Jack S, Murphy J, Varkonyi-Sepp J, et al. Frail2Fit study protocol: a feasibility and acceptability study of a virtual multimodal intervention delivered by volunteers to improve functional outcomes in older adults with frailty after discharge from hospital. BMJ Open. 2023 Mar 16;13(3):e069533.

6.	Kerstiens S, Bender EM, Rizzo MG, Jr., Landi A, Gleason LJ, Huisingh-Scheetz M, et al. Technology-assisted behavioral intervention to encourage prehabilitation in frail older adults undergoing surgery: Development and design of the BeFitMe™ Apple Watch app. Digit Health. 2023 Jan-Dec;9:20552076231203957.

7.	Villa-García L, Davey V, Peréz LM, Soto-Bagaria L, Risco E, Díaz P, et al. Co-designing implementation strategies to promote remote physical activity programs in frail older community-dwellers. Front Public Health. 2023;11:1062843.

8.	Geraedts HA, Zijlstra W, Zhang W, Spoorenberg SL, Báez M, Far IK, et al. A Home-Based Exercise Program Driven by Tablet Application and Mobility Monitoring for Frail Older Adults: Feasibility and Practical Implications. Prev Chronic Dis. 2017 Feb 2;14:E12.

9.	Geraedts HAE, Dijkstra H, Zhang W, Ibarra F, Far IK, Zijlstra W, et al. Effectiveness of an individually tailored home-based exercise rogramme for pre-frail older adults, driven by a tablet application and mobility monitoring: a pilot study. Eur Rev Aging Phys Act. 2021 Jun 21;18(1):10.

10.	Hong J, Kong HJ. Digital Therapeutic Exercises Using Augmented Reality Glasses for Frailty Prevention among Older Adults. Healthc Inform Res. 2023 Oct;29(4):343-51.

11.	Okpara C, Ioannidis G, Thabane L, Adachi JD, Rabinovich A, Hewston P, et al. The Geras virtual frailty rehabilitation program to build resilience in older adults with frailty during COVID-19: a randomized feasibility trial. Pilot Feasibility Stud. 2023 Jul 17;9(1):124.

12.	Wang E, Keller H, Mourtzakis M, Rodrigues IB, Steinke A, Ashe MC, et al. MoveStrong at home: a feasibility study of a model for remote delivery of functional strength and balance training combined with nutrition education for older pre-frail and frail adults. Appl Physiol Nutr Metab. 2022 Dec 1;47(12):1172-86.

13.	Zhu Y, Xie H, Zhang L, Su X-y, Wang H. Effects of Modern Online and Traditional Offline Exercise Programs on the Frailty, Physical function, Emotional, and Social Support on Chinese Community-Dwelling Frail Older Adults: A Randomized Clinical Trail. Nursing Communications. 2021.

14.	Yoo I, Kong HJ, Joo H, Choi Y, Kim SW, Lee KE, et al. User Experience of Augmented Reality Glasses-based Tele-Exercise in Elderly Women. Healthc Inform Res. 2023 Apr;29(2):161-7.

## Supplementary Table 5. Integrated mapping of evidence, user needs, nudging strategies, and intervention components

| Inclusion of behavioral strategies mentioned in the study | Corresponding nudge strategies |
| --- | --- |
| Technical support | Information support nudge, simplification nudge |
| Health education | Information support nudge |
| Goal setting | Pre-commitment nudge |
| Reminder | Reminder nudge |
| Social support | Social norms nudge |
| Feedback | Feedback nudge |
| Visual cues | Environmental cues nudge |
| Progress tracking | Feedback nudge |

Note: Nudge strategy mapping from the scoping review

| Evidence from the scoping review | User needs/preferences from qualitative interviews | COM-B domain | Selected nudging strategies | Resulting intervention components | Logic for matching |
| --- | --- | --- | --- | --- | --- |
| Technical support was commonly included to help participants use digital or home-based exercise systems. | Participants reported concerns about operational complexity and required support in using unfamiliar digital tools. | Physical capability | Information support; simplification; priming | 1.1 Technical guidance | Technical guidance, simplified manuals, videos, and WeChat-based support were used to reduce operational burden, increase familiarity with the system, and support initial engagement. |
| Health education was commonly used to improve exercise-related knowledge and awareness. | Participants preferred personalized, understandable, and practical health knowledge rather than abstract information. | Physical capability | Information support; simplification; social norms; default options | 1.2 Health education | Simplified, illustrated, and automatically delivered health education was used to reduce information burden and make exercise-related knowledge easier to understand and apply. |
| Exercise prescriptions and structured exercise lessons were frequently used in mobile health-based exercise interventions. | Participants preferred exercise lessons that were beneficial, tailored, dynamic, fragmented, and appropriately challenging. | Physical capability | Information support; simplification; default options | 1.3 Exercise lessons | Structured and personalized multicomponent exercise lessons were used to reduce choice overload while preserving tailoring, progression, and perceived relevance. |
| Behavioral strategies in previous interventions often emphasized motivation and long-term benefits. | Participants needed to perceive the future benefits of exercise more concretely. | Psychological capability | Emotional anchoring; framing | 1.4 Future self-visualization | Future self-visualization was used to make long-term health consequences more vivid, emotionally salient, and personally meaningful. |
| Feedback and psychological support were used in previous interventions to support continued participation. | Participants reported barriers related to confidence, fear of exercise, frustration with technology, and competing daily priorities. | Psychological capability | Feedback | 1.5 Psychological adaptation | Regular psychological support and feedback were used to strengthen perceived control, confidence, and coping with exercise-related barriers. |
| Visual cues and environmental prompts were used to support behavior initiation. | Participants needed convenient and visible cues to support home-based exercise. | Physical opportunity | Environmental cues | 2.1 Environmental modification | Home-based cues and environmental arrangement were used to make exercise more visible, accessible, and easier to initiate in daily life. |
| Professional support and feedback were commonly used to increase adherence and safety. | Participants emphasized the need for professional engagement, reassurance, and trustworthy guidance. | Social opportunity | Feedback; expert authority | 2.2 Professional support | Professional consultation, progress review, and video feedback were used to increase trust, perceived safety, and confidence in exercise participation. |
| Social support was commonly included in mobile health and exercise interventions. | Participants expressed a need for encouragement, interaction, and support from peers. | Social opportunity | Social norms; feedback; peer comparison | 2.3 Peer support | Peer interaction and moderator reinforcement were used to create social support, perceived normality, and mutual encouragement. |
| Progress tracking was commonly used to support self-regulation. | Participants needed visible progress and immediate reinforcement to sustain participation. | Reflective motivation | Feedback; environmental cues | 3.1 Self-monitoring | Paper-based logs, star stickers, and system-based progress displays were used to provide visible feedback and reinforce repeated exercise behavior. |
| Goal setting was commonly used to support exercise planning and adherence. | Participants needed clear goals and support in translating intention into action. | Reflective motivation | Precommitment; default options | 3.2 Goal setting | Written precommitment and default exercise goals were used to reduce intention–behavior gaps and support repeated exercise participation. |
| Reminders were commonly used to prompt exercise behavior. | Participants reported competing daily priorities and needed timely prompts to remember and initiate exercise. | Automatic motivation | Reminders; framing; social norms; peer comparison; expert authority | 3.3 Framing reminders | Scheduled reminders and framed messages were used to increase salience, reduce planning neglect, and activate motivational responses through gain, loss, expert, peer, and personalized framing. |
| Social support and Role modeling were used to promote engagement in previous interventions. | Participants responded positively to peer examples and socially meaningful encouragement. | Reflective motivation | Information support; social norms; peer comparison | 3.4 Role modeling | Success stories and recognition of active participants were used to enhance perceived achievability and motivation through similar others’ examples. |
| Gamified elements and reward mechanisms were used in some digital interventions to increase engagement. | Participants showed positive attitudes toward enjoyable, challenging, and rewarding participation formats. | Automatic motivation | Gamification; peer comparison; feedback | 3.5 Gamification design | Points, ranking, “Exercise Star,” and small rewards were used to increase engagement, achievement gratification, and sustained participation. |

## Supplementary Table 6. The preliminary intervention program

| **COM-B Model** | **Nudge Strategy** | **Dimension** | **Intervention Content** | **Intervention Timeline** |
| --- | --- | --- | --- | --- |
| 1. Enhancing the capability of frail older adults to participate | Priming | 1.1 Technical guidance | Provide face-to-face explanations on how to use the home-based exercise system and precautions for older adults, and distribute simple operation manuals. | Weeks 1 |
|  | Information support, Simplification | 1.2 Health education | 1) Frailty Knowledge: Definition, clinical manifestations, relationship with aging, risks, causes, and prevention measures.  2) Scientific Exercise: Physical characteristics of older adults, fitness status, benefits of exercise, importance of multicomponent exercise, safety principles for home-based exercise, fall prevention methods, strategies for managing exercise-related injuries.  3) Chronic Disease Management: Common types of chronic diseases, the impact of frailty on chronic conditions, and the effectiveness of exercise interventions in managing chronic diseases.  4) Digital Literacy: Cybersecurity principles, personal privacy protection measures, strategies to address digital exclusion | Automatic push notifications at 10:00 AM for 12 weeks |
|  | Information support, Default options | 1.3 Exercise lessons | Deliver the Chinese version of VIVIFRAIL multicomponent exercise, including aerobic, resistance, flexibility, and balance training, with four difficulty levels (A, B, C, D; see exercise prescription above). Each session includes an instructional version and a practice version. Exercise videos feature lively background music, positive verbal and textual encouragement, progress bars, countdown icons, and other engaging elements | 3 times/week for 8 weeks |
| 2. Creating opportunities for frail older adults' participation | Simplification | 2.1 Older adults' friendly interface design | 1) Adaptive Design: Large fonts, bright colors, prominent functions, reduced interactions, fewer ads.  2) Information Simplification: Use simple language for health information and exercise guidance; visualize complex information with diagrams.  3) Streamlined Operation: Intuitive, user-friendly interface with minimized steps. | / |
|  | Information support, Precommitment, Default option | 2.2 Intervention Plan | 1) Follow FITT-VP principles (Frequency, Intensity, Type, Time, Volume, Progression) and individual exercise habits. Doctors assess and generate personalized exercise prescriptions and precautions, which are uploaded to the system.  2) Trigger Borg Rating of Perceived Exertion (RPE) after each exercise session.  3) Collaborate with older adults to set stage-specific goals and obtain their commitment: Weeks 1–4: Complete health education articles/videos on time + 3 multicomponent exercise sessions/week. Weeks 5–8: Complete 3 multicomponent exercise sessions/week.  4) Periodic Review: Evaluate commitment adherence and adjust goals as needed. |  |
|  | Feedback, Expert authority | 2.3 Professional support | 1) Provides online consultation services by professionals to address exercise-related queries in real-time.  2) Monthly guided self-assessment of physical changes, with positive reinforcement of exercise adherence.  3) Encourages weekly video submissions of exercise routines for professional form correction and personalized feedback. | Continuous real-time consultation, Monthly progress review, Weekly video feedback |
|  | Social norms, Feedback | 2.4 Peer support | Encourage older adults to share and interact in virtual communities, fostering mutual supervision and encouragement through peer support groups. | / |
| 3. Motivating frail older adults to participate | Feedback, Environmental cues | 3.1 Self-Monitoring | Monitor and visually display exercise duration, health metrics (e.g., heart rate, SpO₂, calorie expenditure), and progress bars showing exercise plan completion. | Real-Time updates |
|  | Framing, Reminders, Social norms, Peer comparison, Expert authority | 3.2 Framing reminders | Standardized message templates with different framing effects, designed by the research team, and personalized reminders triggered by user behavior changes:  1) Risk-Aversion Frame: Highlight potential negative impacts of inactivity.  2) Economic Frame: Emphasize cost savings from reduced medical expenses.  3) Long-Term Benefits Frame: Show 1-, 3-, 5-, and 10-year benefits of regular exercise.  4) Time Perception Frame: Compare exercise time to daily activities.  5) Personalized Frame: Tailor suggestions to health status, preferences, and goals.  6) Expert Authority Frame: Leverage recommendations from experts/institutions for credibility.  7) Social Comparison Frame: Foster intrinsic competition by comparing peer exercise data | Triggered by low participation. |
|  | Informational Support, Social norms, Peer comparison | 3.3 Role modeling | 1) Regularly publish success stories in the WeChat group chat to utilize the Role modeling effect to achieve motivation.  2) Recognize the top scorers and the exercise star of the month in the WeChat group chat, and use the herd mentality to motivate participation. | Monthly |
|  | Gamification, Peer comparison | 3.5 Gamification design | 1) Points ranking: Ranking based on points, initial points of 100 points, 10 points deducted for not completing an exercise, triggering a frame reminder.  2) Exercise Star: The title of “Exercise Star” is awarded to the participants who has trained for the longest period.  3) Reward: Participants who have 100 points at the end of the intervention or who have been awarded the title of “Exercise Star” can get a small gift. | Real-time |

## Supplementary Table 7. Experts' demographic characteristics

| Characteristics | Experts  N (%) |
| --- | --- |
| Age (years) |  |
| ＜40 | 3 (25.0%) |
| 40-50 | 5 (41.7%) |
| ＞50 | 4 (33.3%) |
| Education level |  |
| Bachelor | 2 (16.7%) |
| Master | 6 (50.0%) |
| Doctor | 4 (33.3%) |
| Professional title |  |
| Senior | 8 (66.7%) |
| Middle | 4 (33.3%) |
| Professional field |  |
| Geriatrics, Nursing | 8 (66.6%) |
| Rehabilitation | 2 (16.7%) |
| Software | 2 (16.7%) |
| Work experience (year) |  |
| ＜20 | 4 (33.3%) |
| 20-30 | 6 (50.0%) |
| ＞30 | 2 (16.7%) |

## Supplementary Table 8. Expert suggestions and revisions

| **Category** | **Detailed suggestions** | **Corresponding modifications** |
| --- | --- | --- |
| Adjustments to intervention timing and location | Suggest adopting an "offline-online" phased transition intervention model to avoid over-reliance on mobile devices, which may lead to poor learning outcomes for older adults. | Adjusted the intervention timing and format into three phases: The initial adaptation phase (Weeks 1-2) consisted of three weekly supervised group exercise intervention at community centers, providing both in-person exercise instruction and training on the home-based exercise system to establish baseline competencies. During the transition phase (Weeks 3-4), a hybrid approach was adopted, combining one weekly supervised community session for skill reinforcement with two weekly home-based exercise system sessions to gradually increase participant autonomy. Finally, the consolidation phase (Weeks 5-12) fully transitioned to the home-based exercise system, maintaining three weekly exercise sessions delivered through the mHealth platform with nudge strategies. |
| Clarification between exercise prescription and behavioral intervention strategies | The initial draft mistakenly integrated the "Chinese version of the VIVIFRAIL multicomponent exercise prescription" into the exercise behavior nudge strategy. It is recommended to separate the exercise prescription and behavioral nudge intervention into two distinct tables to clarify their relationship. | Separated the exercise prescription (a scientifically designed exercise program for frail older adults from an exercise physiology perspective) and the exercise behavior nudge intervention (aimed at improving participation and adherence through supportive measures). Both are presented in separate tables for clarity and logical rigor. |
| Supplementation of sub-dimensions within the COM-B model and intervention formats | 1) The current draft only covers the three core COM-B dimensions (Capability, Opportunity, Motivation) without detailing subdimensions (e.g., physical/psychological capability, social/physical opportunity, reflective/automatic motivation). These should be expanded. 2) Specify whether interventions are offline or home exercise system-based. | Added COM-B subdimensions and intervention formats in the final version |
| Addition of a psychological support module | Recommend designing a dedicated psychological support module to enhance older adults’ confidence and motivation in participating in mobile health-based exercise interventions. Include mental health content in health education to help participants address potential psychological challenges when using digital platforms. | Added a psychological support module and integrated mental health education to improve participants’ coping strategies and engagement. |
| Refinement of professional support | Suggest clarifying methods for interpreting assessment results to help older adults recognize the benefits of exercise. | Added guiding examples in the intervention, such as: "*Compared to before, do you find it easier to climb 10 flights of stairs now*?" Affirm their efforts to reinforce exercise adherence. |
| Modification of health education materials | Current health education content should be restructured as an appendix or pre-learning module, as it lacks key nudge theory elements (e.g., behavioral triggers, choice architecture optimization). | Optimized as follows: 1) Health education covers five dimensions: frailty improvement, exercise rationale, chronic disease management, digital literacy, and psychological adjustment (with automated health information). 2) Enhanced salience: Key information is visualized (e.g., illustrations). 3) Peer examples are incorporated to leverage social proof and reduce resistance. 4) Abstract concepts are replaced with actionable steps (e.g., "Aerobic exercise improves cardiorespiratory function" → "Walk from the living room to the balcony 5 times daily"). Habit bundling: Pair exercises with daily routines (e.g., "Do 3 chair stands when the news broadcast theme plays" with illustrations). Risk visualization: Compare inactivity to "a rusty door hinge" (with an image). |
| Optimization of nudge strategies | 1) Introduce environmental nudges to subtly promote exercise. 2) Add a "health age frame" to reframe health messages and highlight exercise importance. 3) Specify nudge reminder methods (e.g., random/sequential triggers). 4) Add personalized feedback nudges tailored to health status and needs. 5) Replace "demonstration comparison" with "Role modeling priming." 6) Expand frame reminder details with concrete examples. | Implemented the following optimizations: 1) Environmental nudges: Suggestions for home exercise setups (e.g., placing equipment visibly with "Daily Exercise" reminder signs). 2) Framing nudges: Added "health age frame" (e.g., "Regular exercise can make your health age 5 years younger than your actual age."). 3) Reminder methods: Default reminders (3x/week) use gain framing, time perception framing, expert authority framing, and social comparison framing; if ineffective, trigger loss aversion, economic benefit, long-term benefit, health age, or personalized framing. 4) Personalized feedback: Adjust frequency based on health status (more frequent for high-need individuals). 5) Renamed "demonstration comparison" to "Role modeling priming." 6) Added detailed examples for each reminder frame (see Table 3) for practicality. |

## Supplementary Figure 1. The PRISMA flow diagram
